# The Association Between Nutritional Status and Muscle Strength of Shoulder, Hip, and Knee, and the Timed Up and Go Test in Older Adults

**DOI:** 10.3390/nu17172850

**Published:** 2025-09-01

**Authors:** Abrar Melebari, Dara Aldisi, Mahmoud M. A. Abulmeaty, Adel Alhamdan

**Affiliations:** Department of Community Health Sciences, College of Applied Medical Sciences, King Saud University, Riyadh 11451, Saudi Arabia; abrarmelebari1@gmail.com (A.M.); daldisi@ksu.edu.sa (D.A.); mabulmeaty@ksu.edu.sa (M.M.A.A.)

**Keywords:** muscle strength, sarcopenia, older adult, malnutrition, functional performance, nutritional status

## Abstract

**Background/Objectives:** This study examined the relationship between nutritional status and muscle strength in the upper and lower large muscles, with a specific focus on shoulder, hip, and knee muscle strength in community-dwelling older adults. It also investigated the relationship between nutritional status and functional performance as measured by the Timed Up and Go (TUG) test. **Methods:** A secondary analysis of a cross-sectional study involving 2045 older adults (aged 60 years or older) from 15 randomly selected primary health care centers in Riyadh was conducted between January 2015 and April 2017. After excluding participants with specific medical conditions, 1741 individuals were included in the analysis. Nutritional status was assessed using the Arabic version of the Mini-Nutritional Assessment (MNA). Handgrip strength (HGS) and large muscle strength (shoulder, hip, knee) were measured using a hydraulic hand dynamometer and the Nicholas Manual Muscle Tester, respectively. The TUG test was also assessed to evaluate physical performance. **Results:** The study revealed that well-nourished older adults had significantly greater upper and lower muscle strength than those who were malnourished or at risk (*p* ≤ 0.001), with males generally exhibiting stronger muscle strength than females. Furthermore, in both genders, older adults showed significant positive correlations between MNA scores and upper and lower muscle strength. Additionally, longer times on the TUG test indicated a higher risk of malnutrition (OR: 1.135, CI: 1.087–1.186; *p* ≤ 0.001). **Conclusions:** this study breaks new ground by being the first to explore the relationship between nutritional status and the muscular strength in the shoulder and hip regions. Findings indicate that muscle strength and physical performance were associated with malnutrition.

## 1. Introduction

Globally, the number of older adults is growing rapidly and may soon outnumber younger people in several countries. Longevity in adults is becoming more prevalent in the developed world, as noted during the initial half of the 20th century [1]. The global population over 60 will almost double, from 12% in 2015 to 22% in 2050 [2]. Saudi Arabia’s older adults will significantly expand in the coming decades. According to projections, the percentage of older adults (≥60 years) comprises about 6% of the total population, and it is expected to increase dramatically to reach around 23% by 2050 [3]. In number, it is expected to increase fivefold, i.e., from 2 million in 2020 to 10.5 million in 2050 [4].

The aging process is associated with physiological alterations, including changes in musculoskeletal, cardiovascular, sensory, and cognitive functions, which can impact nutritional status and overall health [5]. Sarcopenia, defined as the progressive loss of skeletal muscle mass and strength [6], is closely linked to both nutritional status and muscle function, making it highly relevant to our study aim. It significantly affects independence, well-being, and quality of life in older adults [7]. While handgrip strength and chair stand tests are standard diagnostic indicators according to EWGSOP and AWGS criteria [8,9] examining how nutritional status relates to specific large muscle groups (shoulder, hip, knee) provides additional insight into sarcopenia’s functional impact and may aid in early detection and prevention strategies. Global studies found that the prevalence of malnutrition among older adults varies from 13% to 54% [10]. According to several studies, malnutrition is highly prevalent among community-dwelling older adults in Saudi Arabia. For example, a recent cross-sectional study in the Makkah region showed that 13.3% were malnourished and 53.9% were at risk of malnutrition [11]. Another cross-sectional study in Jeddah, conducted by Alzhrani and his colleagues, found that 38.2% of outpatient older adults were malnourished or at risk of malnutrition [12]. In Riyadh city, an extensive population-based cross-sectional study involving 2045 older adults revealed that around 21% of older individuals were identified as being at risk of or suffering from malnutrition [13].

While aging is commonly associated with a decline in muscle mass, its effects are more pervasive, also negatively impacting overall lean body mass and various associated physiological parameters, such as muscle quality, bone mineral density, and metabolic function [6,14]. These comprehensive age-related changes contribute to a significant decrease in muscle strength and overall physical performance, which in turn can profoundly affect older adults’ independence, well-being, and quality of life [7]. Most studies investigating the relationship between nutritional status and muscle strength in older adults evaluated muscle strength via handgrip strength (HGS) [15,16,17,18]. However, the association between nutritional status and strength of other large upper and lower muscle groups, such as shoulder, hip, and knee muscle strength, is limited and has not yet been investigated in community-dwelling older adults. Thus, the primary objective of the present study was to examine the relationship between nutritional status and muscle strength of the upper and lower large muscles. The secondary objective was to investigate the association between nutritional status and the TUG test.

## 2. Materials and Methods

### 2.1. Study Design and Sample Selection

This is a secondary analysis of a cross-sectional study (project number 10-med121902) involving 2045 older adults aged 60 years or older who visited 15 randomly selected primary health care centers (PHCCs) in Riyadh city between January 2015 and April 2017. The original project aimed to evaluate the internal environment of PHCCs and the health status, including nutritional status, muscle strength, and physical function, of older adults visiting these centers. The project was approved by the Ethical Committee at the Ministry of Health, KSA (reference number: 10S/72). Details of the experimental design and sampling of this project have been described elsewhere [13,16,19]. For this secondary analysis, ethical approval was obtained from the Institutional Review Board (IRB) of the College of Medicine at King Saud University, under reference number 23/0924/IRB, dated 19 November 2023. The IRB confirmed that no additional ethical review was required, as the data utilized were fully de-identified and the secondary analysis adhered to the original protocol’s ethical conditions. The IRB confirmed that no additional ethical review was required, as the data utilized were fully de-identified and the secondary analysis adhered to the original protocol’s ethical conditions.

In the present study, a total of 1741 older adults (55.2% male) were extracted after excluding those having neurological disorders, cancer, major musculoskeletal disorders, deformities of body parts, under polypharmacy, hospitalized patients, and those having surgery within three months.

### 2.2. Methodology

Methods for assessing nutritional status, HGS, and the TUG test were described previously [13,16,19]. The Mini-Nutritional Assessment (MNA), a validated Arabic version of the full form, was used to assess nutritional status [20]. For HGS, it was assessed using the Jamar Hydraulic Hand Dynamometer and measured in kilograms [16]. Muscle strength of shoulder, hip, and knee extension, measured in kilograms, was assessed using the Nicholas Manual Muscle Tester (NMMT), which is considered a reliable and valid measure in older adults [21,22,23].

#### 2.2.1. Shoulder Abduction at 0° and Abduction at 90°

The participant was tested while standing or seated, with the shoulder in neutral (0°) and 90° abduction positions. The arm was stabilized, and the NMMT device was calibrated and positioned 3 cm above the wrist joint. The peak force displayed on the NMMT was recorded to quantify shoulder abduction strength at the specified angles.

#### 2.2.2. Hip Abduction and Flexion

The participant was tested in a supine position. The NMMT was positioned 3 cm above the ankle joint. For hip abduction, the right leg was moved laterally away from the midline approximately 30 cm off the table, and the examiner applied a force. In hip flexion, the participant flexed the hip by lifting it upward approximately 20 cm from full extension [24].

#### 2.2.3. Knee Extension

The participant sat with their arms crossed over their chests, their knees and hips bent at a fixed 90-degree angle, and their feet lifted off the ground. The examiner held an NMMT stationary approximately 10 cm above the ankle joint while the subjects applied full force for three seconds during the evaluation [24].

#### 2.2.4. The TUG Test

The TUG test was used to evaluate physical performance, which involved standing from an armchair, walking three meters, returning, and sitting down. The time was measured in seconds with a stopwatch [25].

### 2.3. Statistical Analysis

Data were analyzed using the Statistical Package for the Social Sciences (SPSS) software, version 22 (IBM Corporation, Armonk, NY, USA). Based on normality testing using the Shapiro–Wilk test, it was shown that the data were not normally distributed. Continuous variables of this study were presented as medians and interquartile ranges. The Mann–Whitney test was used to compare the continuous outcomes between the well-nourished and at-risk or malnutrition groups, or between males and females. To investigate the associations among continuous variables, Spearman’s correlation coefficient was employed. In addition, binary logistic regression analysis was performed to assess the impact of upper and lower muscle strength and TUG (as independent variables) on nutritional status (outcome variables), using the malnourished or at-risk malnutrition group as a reference. *p*-values ≤ 0.05 were considered statistically significant.

## 3. Results

### 3.1. Nutritional Status, Upper and Lower Muscle Strength, and Timed Up and Go Test

Table 1 and Table 2 present the association between nutritional status and muscle strength of the upper and lower extremities in older adults of both genders. The results showed that in both genders, the well-nourished group had significantly greater upper and lower muscle strength compared to those classified as malnourished or at risk for malnutrition (*p*-value ≤ 0.001). In addition, as expected, males in both the well-nourished and the malnourished or at-risk malnutrition groups had significantly higher muscle strength than females in the corresponding groups.

Concerning upper muscle strengths, Table 1 indicates that the largest difference between the well-nourished and the malnourished or at-risk-of-malnutrition groups, in percentage, was seen in the shoulder abduction strength at 0° in males (25%). In contrast, the smallest difference was observed in females’ shoulder abduction strength at 90° (5.6%). Regarding lower muscle strength, Table 2 indicates that in males, the difference in knee extension strength (KES) and hip abduction, expressed as a percentage, between the two groups (the well-nourished group and the malnourished or at-risk-of-malnutrition group) was 38.6% and 25.9%, respectively. Conversely, the lowest difference was observed in females’ hip flexion, about 10%.

Table 3 and Table 4 show the correlations between MNA scores, upper and lower muscle strength, and the TUG test in male and female older adults, respectively. Table 3 indicates that in males, there is a significant positive correlation between MNA scores and upper and lower muscle strength. Among muscle strength, the strongest correlations were observed between shoulder abduction at 0° and the following muscle strength: shoulder abduction at 90° (r = 0.834), hip abduction (r = 0.777), and hip flexion (r = 0.746). In addition, KES demonstrated strong positive correlations with hip abduction (r = 0.826) and hip flexion (r = 0.828).

Table 4 indicates that in females, there is a significant correlation between MNA score and upper and lower muscle strength, except with HGS. Among muscle strength, strong correlations were observed between shoulder abduction at 0° and the following lower muscle strength: KES (r = 0.744), hip abduction (r = 0.832), and hip flexion (r = 0.838). KES also demonstrates a strong positive correlation with hip abduction (r = 0.755) and hip flexion (r = 0.744). TUG showed a significant negative correlation with MNA scores and HGS in females only.

### 3.2. Binary Logistic Regression by Nutritional Status

Table 5 presents the binary logistic regression analysis to evaluate the impact of muscle strength and TUG on nutritional status, using the malnourished or vulnerable to malnutrition group as a reference. In the crude model, the results indicated that increased upper and lower muscle strength significantly decreased the OR of being at risk of malnutrition or malnourished. Furthermore, TUG displayed a significant association with nutritional status, in which the increase in the time spent to complete TUG increased the odds of being at risk of malnutrition or malnourished (OR: 1.098, CI: 1.057–1.140; *p*-value: 0.001). After adjusting the model for gender, age, marital status, educational level, income, and BMI, the relationship remained significant (*p*-value: 0.001).

## 4. Discussion

Several studies have assessed the relationship between muscle strength, particularly HGS, KES, and TUG, and nutritional status [24,26,27,28]. However, this is the first study designed to investigate the association between shoulder and hip muscle strength and nutritional status. The study showed that the well-nourished group had significantly higher shoulder and hip muscle strength compared to the group with malnutrition or at risk of developing it. Alotaibi and her colleagues observed higher HGS and KES in the well-nourished group compared to the malnourished group using the short form of the mini-nutritional assessment, which aligns with our findings. This may indicate that weak muscle strength in both upper and lower muscles is associated with malnutrition. Beyond muscle strength, our findings could have implications for broader age-related body composition changes. Fat Mass Index is important, as excess adiposity—especially in sarcopenic obesity—can worsen functional decline [29,30,31]. Total Lean Mass Index is strongly linked to physical performance, making its preservation essential for mobility [32,33]. Bone Mineral Density is closely associated with muscle health, and nutritional impairment can reduce both lean mass and bone density, as shown in premenopausal anorexic patients [34,35]. Finally, the Skeletal Muscle Mass Index is a key diagnostic indicator for sarcopenia and predicts adverse outcomes such as frailty and mortality [9].

We have noticed that the correlation between shoulder and hip strength with MNA is stronger than between HGS and MNA in both genders (Table 3 and Table 4). In both genders, significant correlations were found between muscle strength in the shoulder, hip, and KES with nutritional status. However, concerning HGS, the correlation was significant only in men. Previous studies have indicated a significant correlation between HGS and nutritional status in both male and female inpatients and outpatients [24,36,37]. The TUG test was used to evaluate physical performance. Our results reveal a significant negative correlation between nutritional status and the TUG test, but only in females (r = −0.140). This agrees with a cross-sectional study of older adults, which found that the correlation between the TUG test and the MNA-SF score was significant only in female participants [38]. However, Ramsey and his colleagues observed a significant positive association between malnutrition and the TUG test in both genders (r= 0.37) [27].

Binary regression analysis was performed to evaluate the impact of muscle strength and TUG on nutritional status. The results revealed that increased upper and lower muscle strength and high physical performance decreased the risk of malnutrition. These findings highlight the importance of maintaining adequate nutritional status to preserve muscle strength, including shoulder and hip muscle strength, and physical performance among the aging population. The relationship between muscular strength, hospitalization, and mortality with aging was studied by Guadalupe-Grau et al. (2015) [39]. The study analyzed 1755 adults aged ≥65 years from the Toledo Study for Healthy Aging, examining upper (handgrip, shoulder) and lower limb (knee, hip) maximal isometric strength in relation to 5.5-year mortality and 3-year hospitalization risk. They found that regional muscle strength predicted both outcomes, with gender-specific patterns—hip, shoulder, and knee strength were stronger predictors in women. In contrast, shoulder and handgrip strength were more relevant in men. Importantly, assessing multiple muscle groups had a cumulative effect: each additional weak muscle region increased mortality risk by 45% in women and 25% in men (*p* < 0.05). This supports our multi-muscle group approach as being more informative than single-measure strength assessments [39].

The study’s main limitations include its cross-sectional design, which does not establish causality, and a lack of national representation, as it involved older adults residing in Riyadh, the capital of KSA, which may not accurately represent older populations from different regions of the country. One of the major strengths of the study was its large sample size. Additionally, the random selection of primary health care centers and the sample were taken from Riyadh city, the capital and largest city of KSA, where older citizens from different regions reside.

## 5. Conclusions

This study is the first to explore the association between nutritional status and shoulder and hip muscle strength. The results suggest that weak muscle strength in the shoulder and hip, in addition to low physical performance, are associated with malnutrition. HGS or chair stand tests are currently used as muscle strength cutoff points for diagnosing sarcopenia in the European Working Group on Sarcopenia in Older People and the Asian Working Group for Sarcopenia [6,9]. Sarcopenia is closely related to muscle strength and nutritional status [15,16,17,18]. Thus, studies are needed to determine appropriate cutoff points for muscle strength in shoulder, hip, and knee extension, and to verify the validity of these cutoff points in predicting sarcopenia. These cutoff points can be used as alternative tools to evaluate sarcopenia in individuals who cannot apply HGS or a chair stand test for medical reasons, like partial amputation or paralysis.

## Figures and Tables

**Table 1:** Upper muscle strength of older adults in the study groups.

MNA						
Variable	Gender	Well-Nourished	*p*-Value *	At Risk of Malnutrition or Malnourished	*p*-Value *	*p*-Value **
Shoulder abduction at 0° (kg)	Male	8 (4)	0.001	6 (2.5)	0.002	0.001
	Female	6.10 (3.90)		5.30 (2.40)		0.001
Shoulder abduction at 90° (kg)	Male	6.80 (3.40)	0.001	5.80 (2.60)	0.004	0.001
	Female	5.50 (1.90)		5.20 (2.30)		0.001
HGS (kg)	Male	30 (9)	0.001	27 (11)	0.001	0.001
	Female	20 (8)		18 (12)		0.001

Data are presented as median and IQR. Mann–Whitney U Test. Significance at *p*-value ≤ 0.05. *p*-value * means the difference in muscle strength between males and females within the well-nourished groups and within the malnourished or at-risk malnutrition groups. *p*-value ** means the difference in muscle strength between the well-nourished group and the malnourished or at-risk malnutrition group, within both genders. MNA, mini-nutritional assessment; HGS, handgrip strength.

**Table 2:** Lower muscle strength of older adults in the study groups.

MNA						
Variable	Gender	Well-Nourished	*p*-Value *	At Risk of Malnutrition or Malnourished	*p*-Value *	*p*-Value **
KES (kg)	Male	10.10 (5.90)	0.001	6.20 (5.60)	0.001	0.001
	Female	6.50 (3.80)		5.30 (3.50)		0.001
Hip abduction (kg)	Male	8.10 (3.85)	0.001	6 (2.20)	0.001	0.001
	Female	5.90 (3.05)		5.30 (1.60)		0.001
Hip flexion (kg)	Male	8.20 (3.80)	0.001	6.30 (3.10)	0.001	0.001
	Female	6 (3.15)		5.40 (2.30)		0.001

Data are presented as median and IQR. Mann–Whitney U Test. Significance at *p*-value ≤ 0.05. *p*-value * means the difference in muscle strength between males or females within the well-nourished groups and within the malnourished or at-risk malnutrition groups. *p*-value ** means testing the difference in muscle strength between the well-nourished group and the malnourished or at-risk malnutrition group, within both genders. MNA, mini-nutritional assessment; KES, knee extension strength.

**Table 3:** Correlation matrix between MNA scores and upper and lower muscle strength and timed up-and-go test in male older adults.

Variable	MNA Scores	Shoulder Abduction at 0°	Shoulder Abduction at 90°	HGS	KES	Hip Abduction	Hip Flexion	TUG
MNA scores	-	0.367 ^b^	0.272 ^b^	0.187 ^b^	0.484 ^b^	0.473 ^b^	0.439 ^b^	−0.003
Shoulder abduction at 0°	-	-	0.834 ^b^	0.291 ^b^	0.687 ^b^	0.777 ^b^	0.746 ^b^	0.005
Shoulder abduction at 90°	-	-	-	0.303 ^b^	0.561 ^b^	0.705 ^b^	0.657 ^b^	0.035
HGS	-	-	-	-	0.135 ^b^	0.187 ^b^	0.187 ^b^	−0.090 ^a^
KES	-	-	-	-	-	0.826 ^b^	0.828 ^b^	0.047
Hip abduction	-	-	-	-	-	-	0.874 ^b^	0.039
Hip flexion	-	-	-	-	-	-	-	0.045

MNA, mini-nutritional assessment; HGS, handgrip strength; KES, knee extension strength; TUG, timed up and go. Tested by Spearman’s rho correlation. ^a^ at the 0.05 level, and ^b^ at the 0.01 level.

**Table 4:** Correlation matrix between MNA scores and upper and lower muscle strength and timed up-and-go test in female older adults.

Variable	MNA Scores	Shoulder Abduction at 0°	Shoulder Abduction at 90°	HGS	KES	Hip Abduction	Hip Flexion	TUG
MNA scores	-	0.185 ^b^	0.151 ^b^	0.045	0.218 ^b^	0.190 ^b^	0.188 ^b^	−0.140 ^b^
Shoulder abduction at 0°	-	-	0.794 ^b^	0.075	0.744 ^b^	0.832 ^b^	0.838 ^b^	−0.069
Shoulder abduction at 90°	-	-	-	−0.015	0.685 ^b^	0.791 ^b^	0.784 ^b^	0.030
HGS	-	-	-	-	0.099 ^b^	0.017	0.025	−0.136 ^b^
KES	-	-	-	-	-	0.755 ^b^	0.744 ^b^	−0.006
Hip abduction	-	-	-	-	-	-	0.878 ^b^	−0.045
Hip flexion	-							−0.020

MNA, mini-nutritional assessment; HGS, handgrip strength; KES, knee extension strength; TUG, timed up and go. Tested by Spearman’s rho correlation. ^b^ at the 0.01 level.

**Table 5:** Binary logistic regression for upper and lower muscle strength and the timed up-and-go test according to the mini-nutritional assessment.

Variable	OR ^a^ (95% CI)	*p*-Value	OR ^b^ (95% CI)	*p*-Value
Shoulder abduction at 0°	0.781 (0.741–0.822)	0.001	0.836 (0.789–0.887)	0.001
Shoulder abduction at 90°	0.795 (0.750–0.843)	0.001	0.862 (0.814–0.914)	0.001
HGS	0.941 (0.927–0.954)	0.001	0.953 (0.934–0.971)	0.001
KES	0.831 (0.799–0.864	0.001	0.843 (0.804–0.884)	0.001
Hip abduction	0.799 (0.760–0.841)	0.001	0.834 (0.787–0.883)	0.001
Hip flexion	0.809 (0.770–0.849)	0.001	0.843 (0.796–0.892)	0.001
TUG	1.155 (1.117–1.193)	0.001	1.098 (1.057–1.140)	0.001

^a^ means crude odds ratio with a 95% confidence interval; ^b^ OR adjusted for age (continuous variable), gender, marital status (×4 categories), educational level (×4 categories), income (×5 categories), and body mass index (×4 categories). *p*-value ≤ 0.05 is considered statistically significant. HGS, handgrip strength; KES, knee extension strength; TUG, timed up and go.

## Data Availability

Data are available upon reasonable request to the corresponding author. Data are not publicly available, as they are part of a mega project that requires permission from the funding agency.
